# Evaluation of the cytotoxic, anticancer, and genotoxic activities of *Acacia nilotica* flowers and their effects on N-methyl-N-nitrosourea-induced genotoxicity in mice

**DOI:** 10.1007/s11033-022-07662-0

**Published:** 2022-08-07

**Authors:** Kawthar A. Diab, Maha A. Fahmy, Emad M. Hassan, Sayed A. El-Toumy

**Affiliations:** 1 Genetics and Cytology Department, National Research Centre (NRC), 33 El-Bohouth Street, Dokki, Cairo Egypt; 2 Medicinal and Aromatic Plants Research Department, National Research Centre (NRC), 33 El-Bohouth Street, Dokki, Cairo Egypt; 3 Chemistry of Tannins Department, National Research Centre (NRC), 33 El-Bohouth Street, Dokki, Cairo Egypt

**Keywords:** *Acacia nilotica* flower, Direct action mutagen N-methyl-N-nitrosourea, Cell cycle analysis, Comet assay, Micronucleus, Cytotoxicity, Chromosomal aberration, Mice

## Abstract

**Purpose:**

In this study, two main research objectives were examined: (1) the cytotoxic and anticancer activities of the aqueous methanol extract from *Acacia nilotica* flowers on three human cancer cells, namely lung A549, breast MCF-7, and leukemia THP-1 cells, and (2) the genotoxic effects of *A. nilotica* extract and its influence on DNA damage induced by N-methyl-N-nitrosourea (MNU) in mice.

**Methods:**

Mice were orally treated with *A. nilotica* extract (200, 500, and 800 mg/kg for 4 days) with or without MNU (80 mg/kg intraperitoneally for 24 h).

**Results:**

*In vitro* experiments showed that A549 cells were the most sensitive to *A. nilotica* extract among the tested cell lines. *A. nilotica* extract inhibited A549 cell proliferation by blocking the cell cycle at the G_2_/M phase and accumulating apoptotic cells in the sub-G_0_/G_1_ phase in A549 cells. *In vivo* experiments showed that MNU induced positive and negative genotoxicity in bone marrow cells and spermatocytes, respectively. Negative genotoxicity was observed in *A. nilotica* extract-treated groups only. However, *A. nilotica* extract (800 mg/kg) remarkably increased comet tail formation in bone marrow cells. Unexpectedly, the absence of antigenotoxicity was observed in three cotreated groups with *A*. *nilotica* extract and MNU compared with the MNU-treated group. Astonishingly, cotreatment with MNU and *A. nilotica* extract at a dose above 200 mg/kg remarkably increased micronucleus and comet tail formation in bone marrow cells compared with the MNU-treated group.

**Conclusions:**

*A. nilotica* extract possessed anticancer activity with relative genotoxic effects at high doses.

**Supplementary Information:**

The online version contains supplementary material available at 10.1007/s11033-022-07662-0.

## Introduction

N-methyl-N-nitrosourea (MNU) is the oldest nitroso compound distributed in air, foods, tobacco smoke, plastics, cutting fluids, gasoline, agrochemicals, dyes, cosmetics, and pharmaceutical products. MNU exogenously occurs in air emissions from sludge incinerators and is endogenously constructed in the stomach and bowel by the nitrosation of methyl urea with nitrate [1]. MNU, a cell-disrupting agent, was initially used as an antineoplastic agent to treat L1210 leukemia cells implanted in mice [2]. MNU is used for carcinogenic, mutagenic, teratogenic, and immunosuppressant purposes. MNU may induce malignant tumors in several tissues based on animal species, strain, age, dosage, and route of administration. MNU, a potent direct-action mutagen, reacts with DNA directly through the transfer of the alkyl group to nitrogen and oxygen atoms in the nitrogen bases and phosphate group, causing a wide range of DNA adducts. Among them, N7 and N3 alkylpurines, O6-alkylguanine, and O4-alkylthymine adducts produce wrong nucleotide incorporation during DNA replication and consequently produce errors in RNA transcription [1].

Medicinal plants are considered the primary source of several valuable pharmaceutical drugs and have been used globally as an alternative and complementary medicine. The search for medicinal plants that can modify or counteract the unwanted side effects of mutagenic agents is in high demand [3]. *Acacia*, a large genus of the family Fabaceae (Leguminosae), comprises approximately 1350 species and is cultivated in tropical and subtropical regions in Africa, Asia, Australia, and the Caribbean. *Acacia nilotica*, known as kikar, black babul, and gum Arabic tree, has considerable economic importance represented by gum production, fuel-wood and charcoal production, and livestock feed production [4, 5].

All parts of *A. nilotica*, including leaves, bark, root, seed, fruit, flower, gum, and immature pods, have been used to treat various disorders, including cough, diarrhea, cold, fever, congestion, dysentery, hemorrhoid, gallbladder disorder, sclerosis, tuberculosis, smallpox, leprosy, and menstrual problems [6]. Phytochemically, *A. nilotica* plant is a rich source of phenolics, flavonoids, anthocyanins, saponins, carbohydrates, and proteins [5, 6]. *Acacias* are characterized by their small and fragrant flowers, which are rich in volatile terpenoids. *Acacia* flowers’ aroma is used for the preparation of aromatic products and essential oils, and its fragrance is used in cosmetics and perfumes. The infusion of *Acacia* flowers is used in the preparation of digestive, sedative, analgesic and antirheumatic tea [7]. Many literature reports have almost exclusively focused on the phytochemical, toxicological and biological screening of the leaves, seeds, pod, root, and bark of *Acacia* species [8–13]. However, the chemopreventive and genotoxicological effects of *A. nilotica* flowers are limited. Therefore, this study was conducted to examine the aqueous methanol extract from *A. nilotica* flowers, based on the following research points: (1) cytotoxicity using sulforhodamine blue (SRB) and 4-[3-(4-iodophenyl)-2-(4-nitrophenyl)-2 H-5-tetrazolio]-1,3-benzene disulfonate (WST-1) assays for adherent (lung A549 and breast MCF-7); and suspension (leukemia THP-1) cancer cells, respectively; (2) anticancer effect using cell cycle analysis via flow cytometry; and (3) genotoxicity and its influence on MNU-induced genotoxicity using chromosomal aberration (CAs), micronucleus (MN), and comet assays in mice.

## Materials and methods

**Plant materials, extraction process and isolation**

*A. nilotica* flowers were collected from Upper Egypt in March 2018. The plant specimen was botanically identified and stored in the herbarium at the Department of Botany, Faculty of Science, Cairo University, under voucher number A356. The flowers were defatted with CHCl_3_ (3 x 1 L) and extracted with methanol (CH_3_OH)/ H_2_O (7:3; 5 × 3 L) at room temperature. The combined extracts were filtered, evaporated under reduced pressure and lyophilized (60 g). The dried extract was loaded on a polyamide 6S column chromatography (80 × 3 cm). The column was eluted with H_2_O, and H_2_O-ethanol mixtures of decreasing polarity and 10 fractions (1 L, each) were collected. The main phenolic fractions obtained were combined into four fractions after chromatographic analysis. Fraction A (2.2 g) was fractionated via column chromatography on Sephadex LH-20 with aqueous ethanol (0%- 70%) for elution to give compounds 1 and 3. Fraction B (2.5 g) was subjected to column chromatography on cellulose and *n*-BuOH saturated with H_2_O as an eluent to yield two main subfractions. Each was separately fractionated on a Sephadex LH-20 to yield pure samples 6 and 5. Under the same procedures, fractions C (2.8 g) and D (2.4 g) gave chromatographically pure samples 4, 2 and 7.

## Cell lines and treatment

Lung adenocarcinoma (A549), breast adenocarcinoma (MCF-7), and acute monocytic leukemia (THP-1) were purchased from the American Type Culture Collection (Manassas, VA, USA). Adherent cancer cell lines (A549 and MCF-7) were grown in a modified eagles medium. The suspension cancer cell line (THP-1) was cultured into an RPMI-1640 medium. Cells were maintained in an appropriate medium supplemented with streptomycin (100 mg/mL), penicillin (100 units/mL), and heat-inactivated fetal bovine serum (10%) in a 5% CO_2_ atmosphere at 37 °C. Cultured media and serum were obtained from GIBCO™ (Grand Island, NY, USA).

## SRB cytotoxicity assay

This assay measures the cellular protein content in the viable cells as described previously [14]. Briefly, adherent cells were harvested through trypsinization and cell suspension (5×10^3^ cells/well, 100 µL) was seeded in 96-well plates for 24 h. Aliquots of a 100 µL medium containing different concentrations of plant extract (10, 20, 50, 75, and 100 µg/mL) were added to each well for 72 h. The medium was aspirated and trichloroacetic acid (10%, 150 µL) was added to each well and left for 1 hour at 4°C to fix the cells attached to the bottom of the wells. The supernatant was removed and the microplates were rinsed with distilled water five times and kept for air-drying. The SRB solution (70 µL, 0.4% w/v in 1% acetic acid) were added to each well, in a dark place at room temperature for 30 min. The microplates were rinsed thrice with acetic acid (1%) and allowed to air-dry overnight. The adsorbed SRB was dissolved by adding 10 mM Tris buffer (pH 10.5, 150 µL) to each well and the plate was gently stirred for 10 min on a shaker platform. The absorbance of the wells was measured using a microplate reader at a wavelength of 540 nm.

## WST-1 cytotoxicity assay

This assay is based on the cleavage of the slightly red tetrazolium salt WST-1 to form a dark red formazan dye by cellular mitochondrial dehydrogenases in living cells [15]. Briefly, THP-1 cells were harvested through centrifugation and cell suspension (3 × 10^3^ cells/ well, 50 µL) was seeded into a 96-well plate. After 24 h, cells were treated with an aliquot of 50 µL medium containing different concentrations of plant extract (10, 20, 50, 75, and 100 µg/mL) for 48 h. The WST-1 reagent (10 µL) was added to each well and cells were incubated for an additional hour at 37°C. The optical density (OD) of each well was measured at 450 nm using a microplate reader. The percentage of cell viability was calculated according to the following equation: Cell viability (%) = [(OD of treated cells – OD of blank)/ (OD of control – OD of blank) × 100]. Cytotoxic activity is expressed as IC_50_ (µg/mL), which is the half-maximal inhibitory concentration of the plant extract required to kill 50% of cancer cells and is used to determine drug effectiveness.

### Cell cycle analysis

Cell cycle distribution was performed via flow cytometry as described previously [14]. Briefly, A549 cells (2×10^6^/ 3 mL/six-well plate) were treated with three concentrations of *A. nioltica* (½ IC_50_, IC_50_, and double IC_50_) for 72 h. Treated cells with dimethylsulfoxide and doxorubicin (0.5µM) were used as negative and positive controls, respectively. Cells were harvested and centrifuged at 2000 rpm for 5 min. The supernatant was discarded, and cell pellets were washed twice with phosphate buffer solution (PBS, 2 mL). Cells were fixed overnight in chilled 70% ethanol in a refrigerator at 4°C. The fixed cells were washed and resuspended in PBS (1 mL) containing RNase digestate (400 µg/mL) for 1 h at 37ºC. Subsequently, cells were stained with propidium iodide (10 µg/mL) for 30 min in the dark. Finally, the cells were analyzed immediately for DNA content via flow cytometry using an FL2 (λex/λem = 535/617 nm) signal detector (ACEA Novocyte™ flowcytometer, ACEA Biosciences Inc., San Diego, CA, USA). Cell cycle histograms were analyzed using ACEA NovoExpress™ software (ACEA Biosciences).

## Experimental animals

Mature Swiss male mice of 8–12 weeks old and weighing ~ 25 g were obtained from the animal house colony of the National Research Centre (NRC; Dokki, Cairo, Egypt). Mice were housed in plastic boxes in an air-conditioned room with a temperature of 23 ± 1 °C and relative humidity of 50 ± 20% in a 12-hour light/dark cycle. Mice were provided with a standard balanced pelleted chow diet and chlorinated tap water *ad libitum*. All experimental procedures were performed following the Guiding Principles for the Care and Use of Laboratory Animals approved by the NRC.

### Experimental design

### Ninety mice were equally allocated into nine groups of 10 mice each as follows:

#### Group 1

Negative control group was given distilled water.

#### Group 2

Positive control group was intraperitoneally (i.p) injected with MNU (80 mg/kg, dissolved in distilled water) for 24 h. This dose was selected based on genotoxic studies of MNU in mice [16].

**Groups 3**–**6**: Mice were given orally three doses of *A. nilotica* extract (200, 500, and 800 mg/kg; solubilized in distilled water) alone for four consecutive days.

**Groups 7**–**9**: Mice were treated with *A. nilotica* extract (200, 500, and 800 mg/kg) for 4 consecutive days followed by an i.p injection with MNU (80 mg/kg) after 1 h from the last dose of plant extract.

Mice were killed by cervical dislocation 24 h after the end of the treatment. For chromosomal analysis, bone marrow and testis were collected from half of the animals in each group. For MN and comet assays, bone marrow cells were collected from another half of the animals in each group.

## CAs assay

Mitotic and meiotic CAs were prepared from bone marrow and testis of the same animal, respectively, as described previously in detail [17]. The slides were stained with 10% Giemsa solution in PBS. One hundred cells in mitotic and meiotic metaphases (diakinesis metaphase I spermatocytes) were examined per mouse under a light microscope at 1600× magnification for the presence of CAs.

## MN assay

The bone marrow MN assay was conducted as described previously in detail [17]. The slides were stained using the May-Grünwald–Giemsa staining protocol and examined under a light microscope at ×1600 magnification. Two thousand polychromatic erythrocytes (PCE, immature erythrocytes) were analyzed per mouse for the presence of MN.

## Single cell gel electrophoresis (Comet assay)

The comet assay was conducted in mouse bone marrow under alkaline conditions (pH > 13) as described previously in details [17]. The slides were stained with ethidium bromide and immediately examined at ×400 magnification using an upright fluorescent microscope equipped with a digital camera and green light excitation filter. Two hundred nucleoid were analyzed per animal using comet score™ version 2.0.0.0 (TriTek Corp., Sumerduck, VA, USA). The percentage of DNA in the comet tail (tail DNA) is considered the most reliable parameter to quantify DNA damage.

### Data analysis

Data were analyzed using SPSS version 20 (Statistical Package of Social Science, Armonk, NY, UAS; IBM Corp). Data were checked for normality and the homogeneity of the variance using the Kolmogorov–Smirnov’s and Levene’s tests, respectively. The differences among groups with normal distribution were analyzed via one-way analysis of variance (ANOVA) followed by Tukey honestly significant difference (HSD) test. Results were regarded as significant when *P* < 0.05.

## Results

### Identification of isolated polyphenol compounds

Seven polyphenol compounds were isolated from the aqueous methanol extract from *A. nilotica* flowers. By comparing their chromatographic and spectroscopic data with those reported in the literature [18, 19], these compounds were identified as follows:

**Gallic acid (1)**: ^1^ H-nuclear magnetic resonance (NMR) *δ* (ppm): 6.98 (s, H-2 and H-6).^13^ C-NMR *δ* (ppm): 120.60 (C-1), 108.80 (C-2), 145.50 (C-3), 138.10 (C-4), 145.50 (C-5), 108.80 (C-6), 167.70 (C-7).

**Catechin (2)**: ^1^ H-NMR δ (ppm): 6.72 (*d*, *J* = 1.4 Hz, H-2’), 6.69 (*d*, *J* = 8.1 Hz, H-5’), 6.59 (*dd*, *J* = 8.1 and *J* = 1.4 Hz, H-6’), 5.88 (*d*, *J* = 2.1 Hz, H-6), 5.70 (*d*, *J* = 2.1 Hz, H-8), 4.49 (*d*, *J* = 7.34 Hz, H-2), 3.63 (m, H-3), 2.65 (*eq*., *dd*, *J* = 15.87 and *J* = 5.31, H-4) or 2.35 (*ax*., *dd*, *J* = 15.94 and *J* = 7.86 Hz, H-4). ^13^ C-NMR *δ* (ppm): 81.27 (C-2), 66.63 (C-3), 28.09 (C-4), 156.50 (C-5), 95.50 (C-6), 156.76 (C-7), 94.24 (C-8), 155.67 (C-9), 99.45 (C-10), 130.94 (C-1’), 114.81 (C-2’), 145.16 (C-3’), 145.16 (C-4’), 115.46 (C-5’), 118.82 (C-6’).

**7-Galloyl catechin (3)**: ^1^ H-NMR *δ* (ppm): 7.27 (s, galloyl), 6.95 (d, *J* = 1.8 Hz, H-2′), 6.85 (d, *J* = 7.8 Hz, H-5’), 6.84 (dd, *J* = 9.9 Hz and 1.8 Hz, H-6’), 6.38 (d, *J* = 2.1 Hz, H-6), 6.28 (d, *J* = 2.1 Hz, H-8), 4.71 (d, *J* = 7.5 Hz, H-2), 3.02 (m, H-3), 2.70 (eq., dd, *J* = 16.5 and 8.1 Hz, H-4) or 2.65 (ax., dd, *J* = 16.5 and 8.1 Hz, H-4).^13^ C-NMR *δ* (ppm): 82.21 (C-2), 67.23 (C-3), 28.14 (C-4), 156.31 (C-5), 101.47 (C-6), 150.82 (C-7), 101.19 (C-8), 156.00 (C-9), 106.25 (C-10), 131.22 (C-1′), 114.53 (C-2′), 145.15 (C-3′), 145.04 (C-4′), 115.13 (C-5′), 119.38 (C-5′), 120.43 (galloyl C-1), 109.68 (galloyl C-2), 145.59 (galloyl C-3), 138.76 (galloyl C-4), 145.59 (galloyl C-5), 109.68 (galloyl C-6), 164.63 (-COO-).

**Quercetin 3-*O*-rhanmnopyranosyl (1→6) glucopyranoside (4)**: ^1^ H-NMR, aglycone, *δ* (ppm): 7.57 (1 H, d, *J* = 2.1 Hz, H-2′), 7.54 (1 H, dd, *J* = 9, 2.1 Hz, H-6′), 6.85 (1 H, d, *J* = 9 Hz, H-5′), 6.39 (1 H, d, *J* = 2.1 Hz, H-8), 6.2 (1 H, d, *J* = 2.1 Hz, H-6). Sugar: 5.35 (1 H, d, *J* = 7.5 Hz, H-1″), 4.4 (1 H, d, *J* = 2.0 Hz, H-1′″), 3.16–3.65 (9 H, m, H-2″–H-6″, H-2′″–H-5′″), 1.01 (3 H, d, *J* = 6.3 Hz, CH_3_ of rhamnose). ^13^ C-NMR, aglycone *δ* (ppm): 158.6 (C-2), 135.6 (C-3), 179.4 (C-4), 163.0 (C-5), 100.1 (C-6), 166.5 (C-7), 95.0 (C-8), 159.3 (C-9), 105.5 (C-10), 123.1 (C-1′), 117.7 (C-2′), 145.9 (C-3′), 149.9 (C-4′), 116.1 (C5′), 123.5 (C-6′). Sugar: glucose at 3-position: *δ* (ppm): 104.8 (C-1″), 75.7 (C-2″), 78.2 (C-3″), 71.4 (C-4″), 77.2 (C-5″), 68.6 (C-6″). Rhamnose at 3-position as terminal sugar: *δ* (ppm): 102.4 (C-1′″), 72.1 (C-2′″), 72.2 (C-3′″), 73.9 (C-4′″), 69.7 (C-5′″), 17.9 CH_3_ of rhamnose.

**Quercetin-3-*O*-α-L-rhamnopyranoside (5)**: ^1^ H-NMR, aglycone *δ* (ppm): 7.33 (1 H, d, *J* = 2.0 Hz, H-2′), 7.28 (1 H, dd, *J* = 2.0 and 8.5 Hz, H-6′), 6.9 (1 H, d, *J* = 8.5 Hz, H-5′), 6.42 (1 H, d, *J* = 2.0 Hz, H-8), 6.23 (1 H, d, *J* = 2.0 Hz, H-6). Sugar: *δ* (ppm): 5.29 (1 H, d, *J* = 1.41 Hz, H-1″), 3.16–3.94 (4 H, m, H-2″–H-5″),1.03 (3 H, d, *J* = 6.15 Hz, CH_3_ of rhamnose). ^13^ C-NMR, aglycone: *δ* (ppm): 157.34 (C-2), 134.27 (C-3), 177.79 (C-4), 161.35 (C-5), 98.78 (C-6), 164.34 (C-7), 93.70 (C-8), 156.86 (C-9), 104.16 (C-10), 121.17 (C-1′), 115.52 (C-2′), 145.25 (C-3′), 148.50 (C-4′), 115.72 (C-5′), 120.80 (C-6′). Sugar: *δ* (ppm): 101.87 (C-1″), 70.43 (C-2″), 70.63 (C-3″), 71.25 (C-4″), 70.11 (C-5″), 17.54 (C-6″).

**Quercetin 3-*O*-β-glucopyranoside (6)**: ^1^ H-NMR, aglycone: *δ* (ppm): 7.67 (1 H, dd, *J* = 2.12 and 8.62 Hz, H-6′), 7.53 (1 H, d, *J* = 2.12 Hz, H-2′), 6.82 (1 H, d, *J* = 8.62 Hz, H-5′), 6.4 (1 H, d, *J* = 1.8 Hz, H-8), 6.2 (1 H, d, *J* = 1.8 Hz, H-6). Sugar: *δ* (ppm): 5.37 (1 H, d, *J* = 7.63 Hz, H-1″), 3.28–3.65 (5 H, m, H-2″-H-6″). ^13^ C-NMR, aglycone: *δ* (ppm): 156.80 (C-2), 133.60 (C-3), 177.50 (C-4), 161.60 (C-5), 98.90 (C-6), 164.60 (C-7), 93.80 (C-8), 156.60 (C-9), 104.00 (C-10), 121.60 (C-1′), 115.80 (C-2′), 145.80 (C-3′), 148.80 (C-4′), 116.20 (C-5′), 122.00 (C-6′). Sugar: *δ* (ppm): 101.20 (C-1″), 71.60 (C-2″), 74.40 (C-3″), 70.02 (C-4″), 77.70 (C-5″), 61.50 (C-6″).

**Quercetin (7)**: ^1^ H-NMR *δ* (ppm): 7.69 (1 H, d, *J* = 2.1 Hz, H-2′), 7.57 (1 H, dd, *J* = 2.1 and 8.4 Hz, H-6′), 6.9 (1 H, d, *J* = 8.4 Hz, H-5′), 6.42 (1 H, d, *J* = 1.8 Hz, H-8), 6.2 (1 H, d, *J* = 1.8 Hz, H-6). ^13^ C-NMR *δ* (ppm): 147.50 (C-2), 136.44 (C-3), 176.55 (C-4), 161.43 (C-5), 98.88 (C-6), 164.59 (C-7), 94.05 (C-8), 156.83 (C-9), 103.71 (C-10), 122.66 (C-1′), 116.31 (C-2′), 145.76 (C-3′), 148.40 (C-4′), 115.76 (C-5′), 120.68 (C-6′).

**Effects of*A. nilotica*extract on cell viability**.

In Table 1, *A. nilotica* extract displayed very lower cytotoxicity against THP-1 cells at concentrations of > 100 µg/mL. By contrast, *A. nilotica* extract showed 50% growth inhibition against A549 and MCF-7 cells at concentrations of < 100 µg /mL. Accordingly, A549 cells were more sensitive to *A. nilotica* extract and better candidates for studying molecular cytotoxicity using cell cycle analysis.

**Table 1:** Cytotoxic activity (IC_50_) of *A. nilotica* extract on human cancer cells

Cell lines	IC_50_ (µg/mL)
A549	59.3
MCF-7	96.9
THP-1	> 100

Human cancer cell lines were seeded and incubated with five concentrations of *A. nilotica* extract ranging from 10 to 100 µg/mL in 96-well microplates. The IC_50_ values were evaluated after 72 h (SRB assay for A549 and MCF-7 cells) and 48 h (WST-1 assay for THP-1 cells)

**Effects of A.*nilotica*extract on the cell cycle distribution**.

**Fig. 1:**
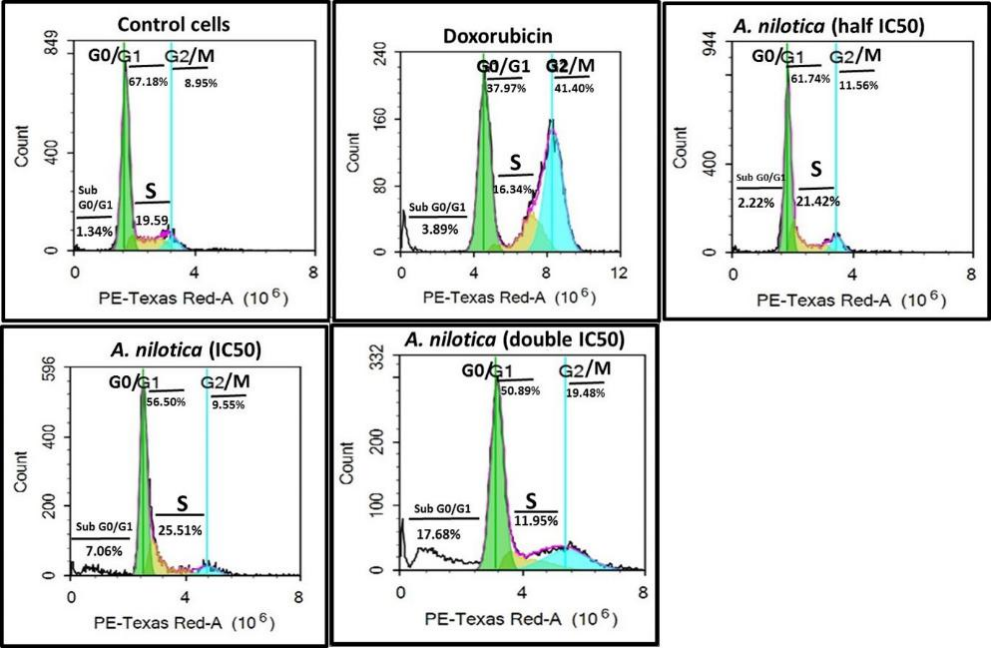
Flow cytometric DNA histogram of A549 cells incubated with three concentrations of *A. nilotica* extract (half IC_50_, IC_50_, and double IC_50_) for 72 h. Representative of one of three similar experiments

To elucidate the molecular mechanism by which *A. nilotica* extract caused growth inhibition in A549 cells, cell cycle distribution was analyzed via flow cytometry (Table 2; Fig. 1). Results showed that treatment with the half-IC_50_ concentration of *A. nilotica* extract had no significant effect on all cell cycle phases compared with control cells. Treatment with IC_50_ and double IC_50_ concentrations of *A. nilotica* extract induced a significant increase in the sub-G_0_/G_1_ apoptotic peak (13.99% and 16.46% vs. 1.34% and 3.12% for negative and positive control cells, respectively). This increase was accompanied by a decrease in cell population in G_0_/G_1_ and S phases. Treatment with *A. nilotica* (IC_50_ and double IC_50_) significantly increased the cell population in the G_2_/M phase (16.29% and 19.91% vs. 9.56% and 46.45% for the negative and positive control cells, respectively).

**Table 2:** Cell cycle distribution in A549 cells after treatment with *A. nilotica* extract within 72 h

Treatment	Sub-G_0_/G_1_	G_0_/G_1_ phase	S-phase	G_2_/M phase
Negative control	1.34 ± 0.03^a^	66.63 ± 0.29^d^	19.62 ± 0.15^bc^	9.56 ± 0.33^a^
Doxorubicin	3.12 ± 0.39^a^	33.94 ± 2.04^a^	16.08 ± 0.67^ab^	46.45 ± 2.55^c^
*A. nilotica* (half IC_50_ )	2.57 ± 0.20^a^	60.77 ± 0.18^ cd^	22.91 ± 1.25^c^	11.02 ± 0.27^a^
*A. nilotica* (IC_50_)	13.99 ± 2.69^b^	53.65 ± 2.74^bc^	15.70 ± 3.82^ab^	16.29 ± 4.36^b^
*A. nilotica* (double IC_50_)	16.46 ± 0.64^b^	51.14 ± 0.22^b^	12.49 ± 0.61^a^	19.91 ± 0.94^b^

Data expressed as mean % ± standard error (S.E). Values with different superscript letters in each column are statistically significantly different from one another as calculated using ANOVA (*P <* 0.05, Tukey HSD test)

## Acute toxicity study

Throughout the 14 day study period, no death was observed in the three groups treated with *A. nilotica* extract at 800, 1600, and 2400 mg/kg. Accordingly, LD_50_ of *A. nilotica* extract was *>* 2400 mg/kg in male mice.

**Effects of*A. nilotica*extract on MNU-induced CAs**.

Tables 3 and 4 summarize the occurrence of CAs induced by treatment with MNU and *A. nilotica* alone or in combination in mouse bone marrow cells and spermatocytes. Oral supplementation with *A. nilotica* extract (200, 500, and 800 mg/kg) did not statistical increase CAs in mouse bone marrow (2.20%, 2.80%, and 3.80%, respectively) and spermatocytes (4.20%, 4.40%, and 6.40%, respectively). Single i.p injection of MNU (80 mg/kg) significantly increased CA levels in mouse bone marrow cells (21.00% vs. 3.40% for the control, *P* < 0.05). However, MNU had no statistical effect on CA levels (4.20% vs. 3.20% for thecontrol, *P* > 0.05) in mouse spermatocytes. Unexpectedly, when three doses *of A. nilotica* extract were given before MNU administration, CA levels did not significantly change in mouse bone marrow cells and spermatocytes compared with the MNU-only treated group in mouse spermatocytes.

**Table 3:** Induction of CAs, MN and comet tail formation after treatment with *A. nilotica* extract and MNU alone or in combination in mouse bone marrow cells

Treatment groups	Metaphases with different type of CAs				Abnormal metaphases		MNPCE/2000 PCE		Tail DNA (%)
	Gap	Br / Fr	Ring	M.A					
	No. (%)	No. (%)	No. %)	No. (%)	No.	Mean % ± S.E	No.	Mean% ± S.E	Mean% ± S.E
Negative Control	9 (1.80)	7 (1.40)	––	1 (0.20)	17	3.40 ± 0.60^a^	51	0.51 ± 0.07^a^	8.01 ± 0.06^a^
MNU (80 mg/kg)	10 (2.00)	74 (14.80)	7(1.40)	15 (3.00)	105	21.00 ± 1.73^b^	385	3.85 ± 0.47^b^	13.97 ± 0.43^c^
Extract LD	6 (1.20)	5 (1.00)	––	––	11	2.20 ± 0.37^a^	90	0.90 ± 0.10^a^	8.21 ± 0.08^a^
Extract MD	7 (1.40)	7 (1.40)	––	––	14	2.80 ± 0.37^a^	107	1.07 ± 0.15^a^	9.00 ± 0.13^a^
Extract HD	8 (1.60)	11 (2.20)	––	––	19	3.80 ± 0.37^a^	139	1.39 ± 0.24^a^	11.61 ± 0.64^b^
Extract LD + MNU	9 (1.80)	54 (10.80)	––	34 (6.80)	97	19.40 ± 0.98^b^	296	2.96 ± 0.14^b^	14.67 ± 0.68^c^
Extract MD + MNU	5 (1.00)	72 (14.40)	––	24 (4.80)	101	20.20 ± 0.92^b^	562	5.62 ± 0.57^c^	15.55 ± 0.48^c^
Extract HD + MNU	4 (0.80)	72 (14.40)	––	21 (4.20)	97	19.40 ± 1.54^b^	740	7.40 ± 0.51^d^	19.21 ± 0.51^d^

LD, low dose (200 mg/kg); MD, middle dose (500 mg/kg); HD, high dose (500 mg/kg); Br/Fr, break (s) or fragment(s); M.A, more than one type of aberrations; MNPCE, micronucleated polychromatic erythrocytes

Ten thousand PCEs (2000 PCEs/ mouse) were counted per group for the presence of MN. One thousand cells (200 cells/mouse) were analyzed using the automatic comet score™. Values with different superscript letters in each column are statistically significantly different from one another as calculated using ANOVA (*P* < 0.05, Tukey HSD test)

**Table 4:** Induction of CAs after treatment with *A. nilotica* extract and MNU alone or in combination in mouse spermatocytes

Treatment groups	No and % of metaphases with different type of CAs				Abnormal metaphases	
	X-Y uni	A-U uni	X-Y + A-U	Br/Fr		
	No. (%)	No. (%)	No. (%)	No. (%)	No.	Mean% ± S.E
Negative Control	13 (2.60)	7 (1.40)	––	––	16	3.20 ± 0.37^a^
MNU	9 (1.80)	7 (1.40)	––	5 (1.00)	21	4.20 ± 0.37^ab^
Extract LD	13 (2.60)	7 (1.40)	––	1 (0.20)	21	4.20 ± 0.80^ab^
Extract MD	12 (2.40)	9 (1.80)	––	1 (0.20)	22	4.40 ± 0.75^ab^
Extract HD	25 (5.00)	7 (1.40)	––	1 (0.20)	32	6.40 ± 0.81^ab^
Extract LD + MNU	19 (3.80)	4 (0.80)	––	(0.20)	24	4.80 ± 0.73^ab^
Extract MD + MNU	27 (5.40)	4 (0.80)	––	––	31	6.20 ± 0.58^ab^
Extract HD + MNU	28 (5.60)	5 (1.00)	2 (0.40)	––	35	7.00 ± 0.95^ab^

LD, low dose (200 mg/kg); MD, middle dose (500 mg/kg); HD, high dose (500 mg/kg); Uni, Univalent; Br/Fr, break(s) or fragment (s). Values with different superscript letters in each column are statistically significantly different from one another as calculated using ANOVA (*P* < 0.05, Tukey HSD test)

**Effects of*A. nilotica*extract on MNU-induced micronucleated PCEs (MNPCEs) and comet tail formation**.

In Table (3), three doses of *A. nilotica* extract did not cause a significant increase in the frequency of MNPCEs (0.90%, 1.07%, and 1.39%, respectively vs. 0.51% for the control) in bone marrow cells. Low and middle doses of *A. nilotica* extract had no statistical effect on the percentage of tail DNA (8.21% and 9.00%, respectively) compared with the control group (Supplementary Fig. 1). However, a high dose of *A. nilotica* extract produced a detectable increase in comet tail formation (11.61% vs. 8.01% for the control).Treatment with MNU considerably increased the MNPCE levels (3.85% vs. 0.51% for the control) and comet tail formation (13.97% vs. 8.01% for the control) in mouse bone marrow cells. Unexpectedly, no inhibitory activity in MNPCEs (2.96%, 5.62%, and 7.40%, respectively) and comet tail formation (14.67%, 15.55%, and 19.21%, respectively) were observed between cotreated groups and the MNU-only-treated group. Surprisingly, cotreatment with middle and high doses of *A. nilotica* extract statistically increased MNPCEs (5.62% and 7.40%, respectively) compared with the MNU-treated group. Cotreatment with MNU and a high dose of *A. nilotica* extract statistically increased comet tail formation (19.21% vs. 13.97% for the positive control).

## Discussion

Generally, medicinal plants contain a mixture of phytochemical constituents that may stimulate or inhibit DNA damage induced by mutagens. In this sense, this study aimed to evaluate two main research objectives: (1) *in vitro* anticancer activity of *A. nilotica* against three human cancer cells namely, A549, MCF-7, and THP-1 cells, and (2) *in vivo* genotoxic activity of *A. nilotica* extract and its effect against MNU in mouse tissues.

*In vitro* experiments showed that *A*. *nilotica* extract exerted moderate cytotoxicity toward A549 (IC_50_ = 59.3 µg/mL) and MCF-7 cells (IC_50_ = 96.9 µg /mL) cells and weak cytotoxic activity toward THP-1 cells (IC_50_ > 100 µg/mL). This finding reflected the fact that cytotoxic criteria of crude extract, based on the IC_50_ value, can be classified into four classes: **(**1) very active potent extract with IC_50_ ≤ 20 µg/mL, (2) moderate active extract with IC_50_ > 20–100 µg/mL, (3) weak active extract with IC_50_ > 100–1000 µg/mL, and (4) extract without cytotoxicity with IC_50_ > 1000 µg/mL [20]. The anticancer activity of natural products depends on several factors, such as (1) the concentration and chemical structure of polyphenols and (2) the origin, morphology, and genomes of cancer cells [21–24]. These observations coincided with previous studies from other parts of *A. nilotica* and other different *Acacia* species [25]. For example, various extracts from *A. nilotica* leaves displayed cytotoxic activity in cervical cancer HeLa cells with IC_50_ values ranging from 28.9 to > 100 µg/mL [26]. Similarly, aqueous ethanol extract from *A. catechu* fruits displayed potent to moderate active cytotoxic activities, with IC_50_ values ranging from 9.7 to 42.8 µg/mL against nine human cancer cell lines, including A549, MCF-7, and THP-1 cells [14]. Moreover, methanol extract from *Acacia. hydaspica* (twigs and leaves) and its fractions ethyl acetate and n-butanol exhibited moderate active cytotoxicity against two breast cancer cells, namely MDA361 and HCC38 cells with IC_50_ values ranging from 29.9 to 75.9 µg/mL [8]. Furthermore, ethanol extract from aerial parts of four *Acacia* species, namely *Acacia salicina*, *Acacia laeta*, *Acacia hamulosa*, and *Acacia tortilis*, possessed moderate active cytotoxicity against four human cancer cell lines, including breast, liver and kidney [9].

Flow cytometric data showed that *A. nilotica* extract (IC_50_ and double IC_50_) markedly reduced A549 cell viability by inducing apoptosis and arresting the cell cycle at the G_2_/M phase. *A. nilotica* polyphenols can bind to DNA and proteins involved in cell cycle progression. Such bindings activate DNA fragmentation, diminish nucleic acids synthesis, and modify the expression of genes implicated in cell cycle arrest at the G_2_/M phase [27, 28]. A similar pattern of results reported by **Sundarraj et al.** [29] found that ethanol extract from *A. nilotica* leaves blocked the cell cycle at the G_2_/M phase in A549 and MCF-7 cells. Several studies have reported that *Acacia* species arrested cell cycle at the G_2_/M phase in leukemia K562 cells [14], breast cancer MDA-MB-231 cells [13], and colon cancer HT-29 cells [12]. Doxorubicin had no pronounced effect on triggering apoptosis in A549 cells compared with *A. nilotica* extract. However, doxorubicin was more prominent in arresting the cell cycle at the G_2_/M phase (46.45%) than *A. nilotica* extract, indicating that apoptosis might not be the core molecular mechanism of cell death in A549 cells treated with 0.5 µM doxorubicin for 72 h. Indeed, various doxorubicin concentrations activate different cytotoxic pathways to induce apoptosis or cell death through mitotic catastrophe [30].

*In vivo* experiments displayed that treatment with MNU significantly increased the occurrence of CAs, MNPCEs, and comet tail formation in mouse bone marrow cells. These findings referred that MUN can methylate DNA at the *O6*-position of guanine, causing GC→AT mutations, DNA double-strand breaks, interstrand cross-links, nonrepaired N-methylpurines, and abasic sites [31–34]. Similarly, MNU produces CAs, MNPCE, and comet tail formation in rodent bone marrow cells, liver, glandular stomach, and peripheral blood lymphocytes of rodents [16, 35–38]. Surprisingly, the absence of genotoxicity was observed in mouse spermatocytes treated with MNU (80 mg/kg) under experimental conditions. This observation was not been mentioned in earlier studies using CA assay in mouse spermatocytes. However, treatment with MNU (75 and 150 mg/kg) produced a positive response in the rat spermatocyte unscheduled DNA synthesis assay [39].

In this study, four days of repeated oral administration of *A. nilotica* extract did not markedly induce the levels of CAs, MNPCEs, and comet tail formation, suggesting its absence of clastogenicity under experimental conditions. However, a high dose of *A. nilotica* extract (800 mg/kg) significantly induced comet tail formation in mouse bone marrow cells. These findings suggested that the comet assay is more sensitive in identifying DNA damage than classical cytogenetic assays. The comet assay measures a variety of DNA damages: DNA strand breaks, DNA-adducts, purinic/apyrimidinic sites or lacking nitrogen base in the DNA region, DNA–protein cross-links, incomplete excision repair sites, and apoptotic nuclei in the cells [40, 41]. These observations revealed that chemical constituents presented in *A. nilotica* extract at high doses showed prooxidant and genotoxic activities in cells. These findings explained that plant-derived antioxidant polyphenols act as prooxidative and antioxidative compounds under certain circumstances in certain tissues, depending on the concentration, metal-reducing potential, chelating behavior, pH, and solubility characteristics [42]. For example, gallic acid, ellagic acid, protocatechuic acid, syringic acid, vanillic acid, caffeic acid, coumaric acid, chlorogenic acid, ferulic acid, myricetin, quercetin, rutin, kaempferol, (+)-catechin, (−)-epicatechin, delphinidin, and malvidin possess dual antioxidant and prooxidant properties [42].Similar to these findings, repeated administration of an aqueous extract from *A. nilotica* roots (500 mg/kg) for 28 days caused hepatotoxicity in rats on biochemical liver function enzymes [10]. Likewise, **Mattana et al.** [43] reported that hot aqueous and ethanol extracts from *A. aroma* leaves (20 mg/mL) exhibit negative and moderate genotoxicity in human blood lymphocytes using the comet assay, respectively.

Contrary to expectations, this study exhibited no antigenotoxicity of *A. nilotica* extract against MNU in mouse bone marrow and spermatocytes. These observations implied that the chemical constituents of *A. nilotica* extract did not prevent or reduce the formation of DNA adducts generated by MNU. Unexpectedly, administration of *A. nilotica* at doses of > 200 mg/kg potentiated the DNA- damaging effect induced by the direct-action alkylating agent MNU in MN and comet assays. These findings can be explained by the fact that many flavonoids intercalate into DNA and inhibit DNA topoisomerases (I and II) by exerting their mutagenic effects [44, 45]. This observation directly agreed with previous literature, which showed that some antioxidant compounds with biphasic nature might exert mutagenic and antimutagenic effects depending on the mutagenic agent and test system used [46, 47]. For example, the absence of antimutagenicity of the ginger extract was observed against DNA damage (comet assay) in blood leukocytes of a mouse model of MNU-induced bladder carcinogenesis [48]. Furthermore, **Arora et al.** [49] examined the antimutagenicity of three extracts from *A. nilotica* bark against direct- and indirect-acting mutagens in the *Salmonella typhimurium* TA 100 strain. The authors found that water and chloroform extracts have no antimutagenicity against direct-acting mutagen (sodium azide), whereas acetone extract was more effective antimutagen against indirect-action mutagen (2-aminofluorene). Furthermore, the antimutagenic activity of *Acacia* species against indirect-action mutagens has been reported in earlier studies. For example, aqueous extracts from the gum, flower, and leaf of *A. nilotica* at doses of 800 mg/kg decreased the frequency of MNPCE and CAs in mouse bone marrow cells induced by indirect mutagen dimethylbenz(a)anthracene [50]. *In vitro* studies showed that different extracts from *A. salicina* leaves reduced comet tail formation induced by H_2_O_2_ in human chronic myelogenous leukemia K562 cells [11].

Given the above data, a possible explanation for the discrepancy between this study and those of earlier investigations might be due to several factors: (1) animal species, gender, age, and tissue response; (2) *Acacia* species and their geographical origin, extraction process, degree of polymerization, and interaction of chemical constituents with each other in the extract; (3) polyphenol concentration in the extract, route of the administration, and duration of treatment; (4) type of mutagen and its mode of action (direct or indirect); and (5) sensitivity or reliability of the genetic marker utilized to detect DNA damage.

## Conclusions

*In vitro* experiments showed that *A. nilotica* extract decreased A549 cell viability by inducing apoptosis and blocking cell cycle progression at the G_2_/M phase. *In vivo* experiments showed no inhibitory activity in the three cotreated groups with *A. nilotica* and MNU compared with the MNU-only-treated group. Surprisingly, *A. nilotica* administration at a high dose potentiated the DNA-damaging effect induced by the direct-action alkylating agent MNU in MN and comet assays. Overall, the dosage use of *A. nilotica* extract may be adjusted for long-term safety.

## Electronic supplementary material

Below is the link to the electronic supplementary material.

Supplementary Material 1

Supplementary Material 2

## Data Availability

All data generated or analysed during this study are included in this published article.
