# Supplementary material for: Evaluation of the cytotoxic, anticancer, and genotoxic activities of Acacia nilotica flowers and their effects on N-methyl-N-nitrosourea-induced genotoxicity in mice
Source: Mol Biol Rep. 2022 Aug 7;49(9):8439–48. doi: 10.1007/s11033-022-07662-0 (PMC9463273; doi:10.1007/s11033-022-07662-0)
Supplement: Supplementary file 1 — Supplementary Material 1 [file 11033_2022_7662_MOESM1_ESM.pdf]

:enago
Author First, Quality First
www.enago.com

ulatus
localize to globalize
www.ulatus.com

voxtab
we are all ears
www.voxtab.com

CERTIFICATE OF EDITING

This is to certify that the paper titled **Evaluation of the cytotoxic, anticancer, and genotoxic activities of Acacia nilotica flowers and their effects on N-methyl-N-nitrosourea-induced genotoxicity in mice** commissioned to us by **Kawthar Abdelaziz Diab** has been edited for English language, grammar, punctuation, and spelling by Enago, the editing brand of Crimson Interactive Inc. under Copyediting/Language editing.

✓ ISO 17100:2015
Translation Service Providers

✓ ISO 27001:2013
Information Security Management System

✓ ISO 9001:2015
Quality Management System

Issued by:
Enago, Crimson Interactive Inc.
160, Greentree Dr, Ste 101 street,
Dover City, Kent, Delaware, 19904
Phone: +1-302-498-8358

Disclaimer : The intent of the author's message has been preserved during the editing process. The author is free to accept or reject our changes in the document after reviewing our edits. This certificate has been awarded at the time of sharing the final edited version (full file or sections of the file) with the author. Enago does not bear any responsibility for any alterations done by the author to the edited document post **20 May 2022**.

**Japan**	www.enago.jp, www.ulatus.jp, www.voxtab.jp	**Russia**	www.enago.ru
**Taiwan**	www.enago.tw, www.ulatus.tw	**Arabic**	www.enago.ae
**China**	www.enago.cn, www.ulatus.cn	**Turkey**	www.enago.com.tr
**Brazil**	www.enago.com.br, www.ulatus.com.br	**S. Korea**	www.enago.co.kr
**Germany**	www.enago.de	**Global**	www.enago.com, www.ulatus.com, www.voxtab.com

About Crimson:
Crimson Interactive INC is one of the world's leading academic research support services. Since 2005, we've supported over 2 million researchers in 125 countries with their publication goals.

JAT
ata American Translators Association
Society for Scholarly Publishing
American Medical Writers Association
EASE
