# Supplementary material for: Evaluation of the cytotoxic, anticancer, and genotoxic activities of Acacia nilotica flowers and their effects on N-methyl-N-nitrosourea-induced genotoxicity in mice
Source: Mol Biol Rep. 2022 Aug 7;49(9):8439–48. doi: 10.1007/s11033-022-07662-0 (PMC9463273; doi:10.1007/s11033-022-07662-0)
Supplement: Supplementary file 2 — Supplementary Material 2 [file 11033_2022_7662_MOESM2_ESM.docx]

**Evaluation of the cytotoxic, anticancer, and genotoxic activities of *Acacia nilotica* flowers and their effects on N-methyl-N-nitrosourea-induced genotoxicity in mice**

**Kawthar A. Diab^1,*^, Maha A. Fahmy^1^, Emad M. Hassan^2^, and Sayed A. El-Toumy^3^**

1. *Genetics and Cytology Department, National Research Centre (NRC), 33 El-Bohouth Street, Dokki, Cairo, Egypt, E. mail:* [*kawthar_diab@yahoo.com*](mailto:kawthar_diab@yahoo.com)
2. *Medicinal and Aromatic Plants Research Department, National Research Centre (NRC), 33 El-Bohouth Street, Dokki, Cairo, Egypt*
3. *Chemistry of Tannins Department, National Research Centre (NRC), 33 El-Bohouth Street, Dokki, Cairo, Egypt*

Supplementary Figure (1): Comet tail formation in mouse bone marrow cells showing (a) control cells; (b) MNU-only-treated cells; (c,d,e) cells treated only with *A. nilotica* extract at doses 200, 500, and 800 mg/kg, respectively; (f,g,h) cells treated with MNU plus low, middle or high doses of *A. nilotica* extract, respectively

*
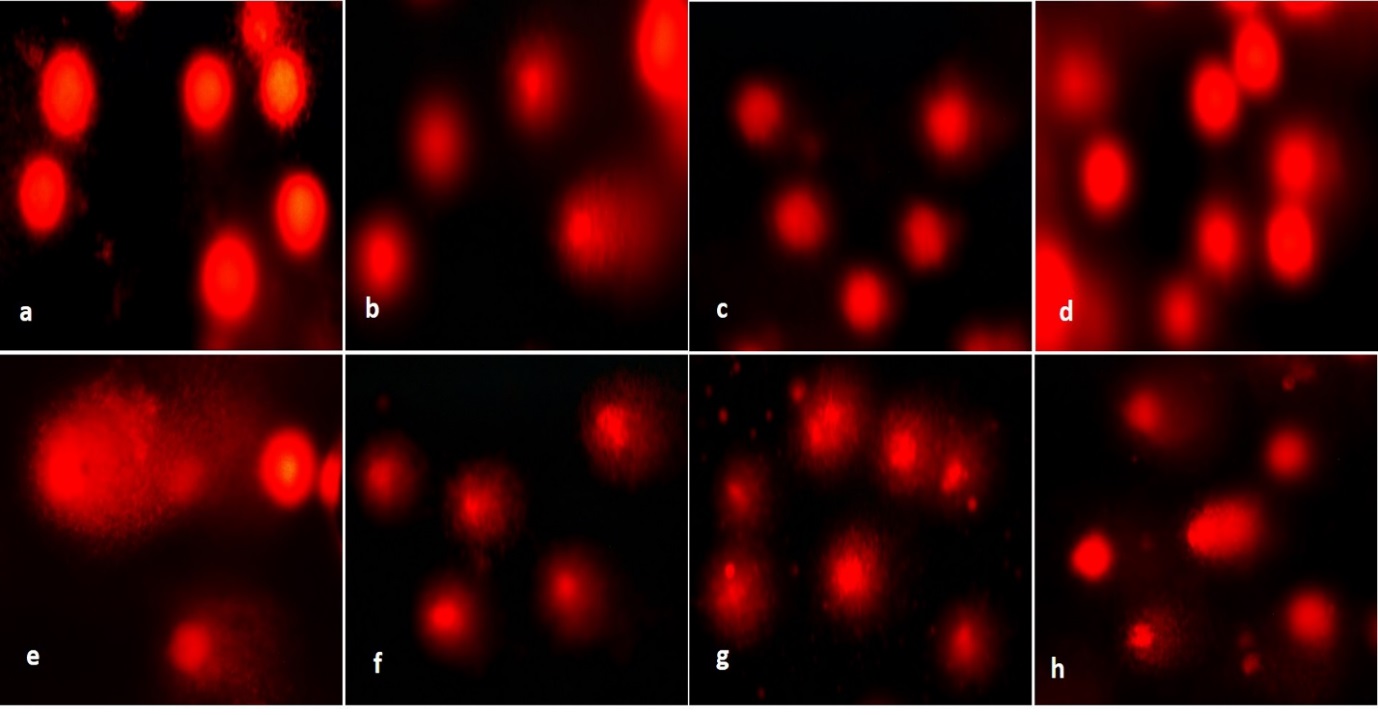
*
